# A Counsel of Despair

**Published:** 1891-02-14

**Authors:** 


### The Doctor's Armchair.

### A COUNSEL OF DESPAIR.

"I hate to see a doctor riding on the back of a policeman," said one of the most popular and successful journalists of the day to the writer. What he meant was that he feels angry when he sees or hears of doctors denouncing everybody as base and worthless who do not happen to say exactly as they say, to do exactly as they do, and generally to conform to professional rules. But there are things to which the ordinary man of physic thinks no practitioner possessed of due self-respect ought to submit. One of these is the case of the man who has studied medicine in the regular way for a longer or a shorter time, but who has failed to pass the examinations required for the acquisition of a legal diploma. Such a man will often begin practice as if he were regularly qualified, and will pay a regularly-qualified practitioner a certain sum annually for the use of his name. Thus, Mr. Smith, who has no degree, will put up a brass plate with the name of Dr. Brown upon it—Brown having a degree. If any legal questions arise which have to be settled in court, Smith the unqualified will play the part of Brown's assistant, and Brown the qualified will give a halo of legality to that which is really illegal, and may be felonious. By a convenient arrangement of this kind the unqualified Smith may secure a nice little income of perhaps seven or eight hundred a-year, whereas his true market value as an unqualified assistant is not more than seventy or eighty. The qualified Brown may pocket, perhaps, a hundred a-year, for which he does nothing at all; and he is really the greater rascal of the two.

When the doctor mounts the policeman's back his object, for the most part, is to put a little skid upon the wheels of such nefarious gentlemen as Smith and Brown; and we cannot but confess our entire sympathy with the fully-qualified medical man when, like a knight errant of old, he rides forth on the back of the representative of law to do battle for his own interests, and for the honour of his profession. We submit that every man has not only the right, but is charged with the duty of defending his own interests; and the doctor would be both a fool and a coward who should neglect to take up his duty boldly when occasion requires. Besides, that doctor, or that association of doctors, which breaks up a partnership in trickery, such as we have described, by means of the policeman, renders a service to public morals. We do not ask any credit for him on this account, because we are aware that his motive is principally or solely one of self-interest. But none the less are we glad to see transparent knavery knocked on the head by a side blow, exactly as we see with pleasure a railway accident avoided, even though the "points" be accidentally put in the right direction by a child who happens to be playing with them.

But it is possible to carry zeal to excess, even in a good cause. There are occasions when sympathy and pity are better than policemen. The writer knows of properly-qualified practitioners who have done in middle life what they would never have dreamt of doing while they were still young and hopeful men. When they began practice their hearts were filled with ideals both worthy and high. They had some little success; and in the confidence of brightening prospects they ventured to marry. Children were born quickly; the health of a wife, unaccustomed to small means and large expenses, drooped and grew feeble. The small and stifling rooms in the narrow and smoky street, called for sea air for children and wife alike. Irregular hours and irregular meals; many patients, but small fees; repeated clangings of the night-bell, and tedious winter hours spent in fireless bed-rooms, for remuneration of the paltriest kind—these have reduced the once robust doctor to a thin, grey, and prematurely aged man before middle life was reached.

Many a man, brought to such a stage as this, has had to choose between giving up his present work at once, and seeing his wife and children droop and die before him, and feeling his own health giving way, quite as surely as theirs. He has chosen to sell his practice, to realise a few hundred pounds, and to betake himself for five or six imperative months to the seaside for rest and fresh air. But at the end of the months, or the year, of rest, destiny has had to be faced again. A practice must be bought or made! Which shall it be? The needy man with his diminished hundreds fears to buy a practice lest he should prove an unsuitable successor, and so find himself burdened with a costly house, but without an income, and with his ready money all spent. He elects, therefore, to try to make a practice; and puts up his door plate and red lamp at a corner house in a suburb of London or other large town. To that doctor and his wife and four or five children the critical moment in life's tragedy has now come. If one or two patients of the right kind consult him, and if he treats them successfully, a tide may rise in his favour which will speedily carry him on to, at least, a moderate competency. But the right patients do not always come; on the contrary, they very seldom come. The patients do not come; but the money flies away; pound by pound at first, for the doctor makes a respectable appearance, hoping thereby to attract patients; shilling by shilling, a little later, because money and hope are alike sinking, and the tide of success is still ebbing further and further from the shore.

Then comes a grim temptation! "What," says the doctor to himself, "would another man do, if he had skilful fingers, a willing heart, and a wife and children who saw starvation peering out of the surrounding darkness? Would he sit still and do nothing? He would up, and seek work for himself, like a man! Why may not I?" Then he thinks that if he were to send round a modest card in the neighbourhood, and to offer to do his work punctually and well, and, above all, at low fees, somebody would seek his services, and so ruin and starvation would be averted. It is a "counsel of despair"! But who shall wonder if, pressed by such temptation, the once high spirited medical student, and high-purposing young practitioner should yield to the voice of the tempter? Even if the card be sent round, it is a hundred to one against its answering the purpose for which it is intended. Its most likely result will be to shut the door of every respectable house in the neighbourhood firmly and finally against the sender. Let no man, therefore, whatever his straits, yield to this peculiar temptation! Relief must be sought, we grant; but there are other ways of relief than this: one of them must be chosen.

But though we have thus counselled some of our weaker brethren against seeking a way of safety, which is, in truth, not a way of safety but of danger, we venture to lift up a protesting hand against those successful practitioners who are always so resolute to crush and destroy a brother who yields to this temptation. Leave him alone, you who are more prosperous! Write no bitter letters to the professional papers! Is the man not humiliated enough by his own act? What harm can he do you? He has sunk too low to have the power of injuring anybody, except, perhaps, himself and his family. Pity him; be sorry for him; and if you can help him up even a single step of the ladder of self-respect again, help him. If anything deserves to be called by the terrible word "damnable," it is the wolf-like readiness of one professional man to turn upon another in his fall and to tear his heart out.

